# Multilineage Differentiation Potential of Equine Adipose-Derived Stromal/Stem Cells from Different Sources

**DOI:** 10.3390/ani13081352

**Published:** 2023-04-15

**Authors:** Hannah J. Stage, Susanne Trappe, Katharina Söllig, Dagmar S. Trachsel, Katharina Kirsch, Cornelia Zieger, Roswitha Merle, Jörg R. Aschenbach, Heidrun Gehlen

**Affiliations:** 1 Equine Clinic, Surgery and Radiology, Department of Veterinary Medicine, Freie Universität Berlin, Oertzenweg 19b, 14163 Berlin, Germany; heidrun.gehlen@fu-berlin.de; 2 Institute of Veterinary Physiology, Freie Universität Berlin, Oertzenweg 19b, 14163 Berlin, Germany; susanne.trappe@fu-berlin.de (S.T.); katharina.soellig@fu-berlin.de (K.S.); kkirsch@atb-potsdam.de (K.K.); joerg.aschenbach@fu-berlin.de (J.R.A.); 3 Clinical Unit of Equine Internal Medicine, Department for Companion Animals and Horses, University of Veterinary Medicine Vienna, Veterinärplatz 1, 1210 Vienna, Austria; dagmar.trachsel@vetmeduni.ac.at; 4 Institute of Veterinary Pathology Department of Veterinary Medicine, Freie Universität Berlin, Robert-von-Ostertag-Straße 15, 14163 Berlin, Germany; cornelia.zieger@fu-berlin.de; 5 Institute for Veterinary Epidemiology and Biostatistics, Department of Veterinary Medicine, Freie Universität Berlin, Königsweg 67, 14163 Berlin, Germany; roswitha.merle@fu-berlin.de

**Keywords:** equine, mesenchymal stromal cells, adipose tissue, proliferation, trilineage differentiation, cardiomyocyte-like cells

## Abstract

**Simple Summary:**

Adult multipotent mesenchymal stem/stromal cells (MSCs) have the ability to self-renew and differentiate into various cell types. MSCs, especially those derived from adipose tissue (ASCs), are easily available and abundant. In order to expand the current knowledge of clinically relevant properties of ASCs obtained from equine abdominal, retrobulbar and subcutaneous adipose tissue using explantation and collagenase digestion, the study’s aim was to investigate the proliferation and multilineage differentiation potential of equine ASCs in vitro. ASCs showed a high proliferation and adipogenic, osteogenic and chondrogenic differentiation potential, whereby a significant effect of the tissue localization and isolation procedure was detected in the case of adipogenic and osteogenic differentiation. However, equine ASCs were refractory to activin A, bone morphogenetic protein-4 and Dickkopf-1-induced upregulation of cardiomyogenic marker expression, characteristic for in vitro cardiomyogenesis. Follow-up studies are required to further investigate the effect of isolation methods and tissue sources on clinically relevant multilineage differentiation potential in equine MSCs. Moreover, the use of stem cells with higher pluripotency should be considered for in vitro cardiomyogenesis in further studies.

**Abstract:**

The investigation of multipotent stem/stromal cells (MSCs) in vitro represents an important basis for translational studies in large animal models. The study’s aim was to examine and compare clinically relevant in vitro properties of equine MSCs, which were isolated from abdominal (abd), retrobulbar (rb) and subcutaneous (sc) adipose tissue by collagenase digestion (ASCs-_SVF_) and an explant technique (ASCs-_EXP_). Firstly, we examined proliferation and trilineage differentiation and, secondly, the cardiomyogenic differentiation potential using activin A, bone morphogenetic protein-4 and Dickkopf-1. Fibroblast-like, plastic-adherent ASCs-_SVF_ and ASCs-_EXP_ were obtained from all sources. The proliferation and chondrogenic differentiation potential did not differ significantly between the isolation methods and localizations. However, abd-ASCs-_EXP_ showed the highest adipogenic differentiation potential compared to rb- and sc-ASCs-_EXP_ on day 7 and abd-ASCs-_SVF_ a higher adipogenic potential compared to abd-ASCs-_EXP_ on day 14. Osteogenic differentiation potential was comparable at day 14, but by day 21, abd-ASCs-_EXP_ demonstrated a higher osteogenic potential compared to abd-ASCs-_SVF_ and rb-ASCs-_EXP_. Cardiomyogenic differentiation could not be achieved. This study provides insight into the proliferation and multilineage differentiation potential of equine ASCs and is expected to provide a basis for future preclinical and clinical studies in horses.

## 1. Introduction

Since the discovery by Friedenstein et al., who first isolated precursor cells from bone marrow in 1968 [1,2], multipotent mesenchymal stem/stromal cells (MSCs) have been studied by many different research groups to this day. MSCs have the ability to self-renew, can differentiate into distinct cell types of mesodermal [3,4,5] and ectodermal origin [6,7] and exhibit a paracrine effect and a regenerative potential [8,9]. These characteristics justify the use of MSCs in various preclinical and clinical studies and make MSCs a promising cell source. In addition to their regeneration ability of tissues, the use in drug testing and in the development of disease models should also be mentioned [10,11,12]. This is based on the identification of appropriate cell sources and the in vitro characterization of the cells harvested [13,14]. MSCs can be isolated from a variety of tissues, including, inter alia, tendon tissue [15,16], synovial fluid [17] and membrane [18,19], peripheral blood [20,21] and adipose tissue [15,16,17,22,23], in addition to bone marrow [1,2]. MSCs from adipose tissue (adipose tissue-derived stem/stromal cells, ASCs) proved to be particularly suitable for use in in vitro models due to their existence in large quantities, large tissue availability [24,25,26] and high proliferation and differentiation potential [14]. ASCs are usually obtained from the easily accessible white adipose tissue (WAT) using stromal vascular fraction (SVF) after collagenase digestion [15,17,27,28,29] or by an explant technique (EXP) [29,30,31]. The identification of the cells is carried out in accordance with the minimum criteria for human MSCs established by the International Society for Cellular Therapy (ISCT), which includes examination of the surface marker profile, the trilineage differentiation potential and the ability to adhere to plastic [32]. The use of various isolation and cultivation methods has a significant influence on cell yield and characteristics of MSCs, i.e., the proliferation and differentiation ability [33,34,35]. The multilineage differentiation potential was investigated in various studies, which included, in particular, the examination of the differentiation potential into adipogenic, osteogenic and chondrogenic lineages (classic trilineage differentiation) [4,23,32,36]. In recent years, the osteogenic, chondrogenic and tenogenic differentiation has shown clinical relevance in equine medicine, whether it is for the treatment of osteoarthritis [37,38], tendon and ligament injury [39,40] or for bone fracture repair in clinical research [41,42,43]. However, the in vitro adipogenesis has currently no clinical relevance in horses. In contrast, in human medicine, adipogenesis models are used in different clinical areas, e.g., for research into diabetes, cardiovascular diseases and mental disorders [44]. The differentiation ability of MSCs into cardiomyocyte-like cells (CLCs) has also been described in certain species [5,45,46,47,48,49], but not for horses, for which there is a lack of evidence so far. For differentiating MSCs into CLCs in vitro, but also embryonic (ESCs) and induced pluripotent stem cells (iPSCs), the induction protocols described in literature often make use of the signaling pathways and transcription factors of embryonic cardiomyogenesis [46,50,51]. These include, in particular, the transforming growth factor (TGF)β/bone morphogenic protein (BMP) [52] and Wnt/β-catenin signaling pathways [53,54]. Further cardiomyogenic differentiation strategies are also described, such as growth factors, small molecules and mechanical and electrical stimulation [55]. In vitro cardiomyogenesis is also of clinical relevance. For example, myocard ischaemia models in rodents have shown that MSCs leads to myocardial regeneration after transplantation [56,57]. Initial clinical studies in humans also exist [58].

In order to be able to use MSCs in a clinical context or for disease models (research into the development of diseases, drug testing, etc.), knowledge of the cultural behavior of cells and proliferation and multilineage differentiation potential in vitro provides an important basis. Therefore, the present in vitro study had the following aims: equine MSCs, which are isolated from abdominal (abd), retrobulbar (rb) and subcutaneous (sc) adipose tissue using the EXP and SVF isolation methods, are investigated with regard to their proliferation potential and further examined regarding their trilineage differentiation potential into adipogenic, osteogenic and chondrogenic lineage. It was hypothesized that equine MSCs behave equally, regardless of the isolation method, and differ from each other depending on the tissue source used. Furthermore, based on the unsuccessful induction of cardiomyogenic differentiation using 5-azacytidine (5-AZA) in our previous study [59], a different protocol for cardiomyogenic differentiation relying on BMP, activin (Act) A and Dickkopf (DKK)-1 was tested in the present study on equine ASCs.

## 2. Materials and Methods 

### 2.1. Sample Collection, Isolation and Cultivation of Equine ASCs 

Sample collection and isolation of equine ASCs have already been published in our previous study [59]. In brief, equine abd, rb and sc adipose tissue from *n* = 16 horses (mares, geldings, stallions; median age [interquartile range]: 13.5 [18.0] years) was harvested after euthanasia at the Equine Clinic: Surgery and Radiology, Department of Veterinary Medicine, Freie Universität (FU) Berlin or slaughter in commercial slaughterhouses up to a maximum of 8 h post mortem. Sampling took place in accordance with the German law of animal welfare and was communicated to the responsible authorities, the Landesamt für Gesundheit und Soziales (LAGeSo) Berlin (StN 008/20–IV C 10).

ASCs were harvested from abd, rb and sc adipose tissue using EXP and SVF techniques as described in Trachsel et al. [59]. Briefly, cell harvest with the SVF method relied on classical collagenase digestion. In the EXP method, 2 × 2 × 2 mm small tissue pieces were placed into 12-well culture plates (tissue culture test plate 12, TPP Techno Plastic Products AG, Trasadingen, Switzerland). After ASCs-_EXP_ grew out into the surrounding, adipose tissue pieces were removed. In both EXP and SVF procedures, adherent precursor cells were cultured in basal medium (B-M), a Dulbecco’s Modified Eagle’s Medium (DMEM) with high glucose (4.5 g/L; Life Technologies GmbH, Karlsruhe, Germany), supplemented with 20% fetal bovine serum (FBS; Sigma Aldrich, Taufkirchen, Germany), 1% amphotericin B (2.5 μg/mL; BioWest, Riverside, CA, USA), 1% penicillin-streptomycin (100 U/mL and 100 μg/mL, respectively; Sigma Aldrich, Taufkirchen, Germany) and HEPES (15 mM; Carl Roth GmbH + Co. KG, Karlsruhe, Germany) under standard culture conditions (37 °C, 5% CO_2_ and a humidified atmosphere). The medium was changed every two to three days. After first passaging, FBS content was reduced to 10% (expansion medium, E-M). The cells were multiplied up to passage (P) 2 or 3 and stored in liquid nitrogen until performance of the experiments described hereafter.

### 2.2. Proliferation Assay

ASCs-_EXP_ and ASCs-_SVF_ of the abd, rb and sc localizations from *n* = 3 horses were included in the experiment. On day zero, 30,000 cells/well of P3 from each source and isolation procedure were seeded in 12-well cell culture plates. According to the protocol of Alipour et al. [60], cells were detached daily after 24 h of cultivation from three wells with Trypsin-EDTA (Trypsin-EDTA solution, 0.25% Trypsin/0.02% EDTA, Sigma Aldrich, Taufkirchen, Germany) and were counted in duplicate using the automated cell counter TC20^TM^ (Bio-Rad Laboratories GmbH, Feldkirchen, Germany). Population doubling time (PDT) was determined using the formula PDT = (T−T0) lg2/(lgNt–lgN0) according to Alipour et al., where T is the harvest time of culture, T0 the starting time of culture, and Nt and N0 are the cell count of harvest and cell count of seeding, respectively [60].

### 2.3. Trilineage Differentiation Potential

To investigate the trilineage differentiation potential, ASCs-_EXP_ and ASCs-_SVF_ of abd adipose tissue and ASCs-_EXP_ of rb and sc adipose tissue were initially expanded to P5 or P6 and then induced to adipogenic, osteogenic and chondrogenic differentiation in two experimental runs (*n* = 6). In all experiments, negative controls (NC) were included. The medium was changed every two to three days. In the case of adipogenic and osteogenic differentiation, cell culture vessels were coated with 2 µg/cm^2^ murine laminin (Sigma Aldrich, Taufkirchen, Germany) in order to avoid cell detachment as far as possible, which was observed in Trachsel et al. due to the absence of any coating [59]. To ensure reproducibility, experiments were carried out in duplicate.

#### 2.3.1. Adipogenic Differentiation

For adipogenic differentiation, 20,000 cells/cm^2^ were seeded in 25 cm^2^ cell culture flasks (5 × 10^5^ cells/25 cm^2^) and 12-well cell culture plates (38,000 cells/well) in duplicate. At 70–90% confluence, standard culture medium was replaced with adipogenic induction medium (day 0) and changed into adipogenic differentiation medium according to Jurek et al. on day 3 [61]. The adipogenic induction medium contained DMEM (Life Technologies GmbH, Karlsruhe, Germany), supplemented with 10% FBS (Sigma Aldrich, Taufkirchen, Germany), 10 mM glucose (Carl Roth GmbH + Co. KG, Karlsruhe, Germany), 100 U/mL and 100 µg/mL penicillin-streptomycin, respectively (Sigma Aldrich, Taufkirchen, Germany), 2.5 µg/mL amphotericin B (BioWest, Riverside, CA, USA), 15 mM HEPES (Carl Roth GmbH + Co. KG, Karlsruhe, Germany), 10 µM biotin, 3 µg/mL insulin, 0.3 µM dexamethasone, 0.1 mM 3-isobutyl-1-methylxanthin (IBMX) and 10 µM rosiglitazone (all from Sigma Aldrich, Taufkirchen, Germany). When adipogenic differentiation medium was used, the last three additives mentioned were replaced by 227 µM ascorbic acid (Sigma Aldrich, Taufkirchen, Germany) and 10 µL/mL bovine serum-lipids (BSL; EX-CYTE, Sigma-Aldrich, Taufkirchen, Germany). Adipogenic differentiation medium was used until days 7 or 14, with medium changes every two to three days.

##### Glycerol 3-Phosphat Dehydrogenase Assay

On day seven, cells in 25 cm^2^ cell culture flasks were washed, trypsinized and centrifuged at 500× *g* at 4 °C for 5 min due to the large lipid droplet formation. The cell pellet was resuspended in Dulbecco’s phosphate-buffered saline (DPBS) without calcium and magnesium (DPBS w/o Ca^2+^/Mg^2+^, Sigma Aldrich, Taufkirchen, Germany). A live/dead staining with 0.4% trypan blue solution (Sigma Aldrich, Taufkirchen, Germany) followed, and cells were counted using an automated cell counter (TC20^TM^; Bio-Rad Laboratories GmbH, Feldkirchen, Germany). After a second centrifugation step (500× *g*, 4 °C, 5 min), further processing followed the GPDH assay kit protocol (Glycerol-3-Phosphate Dehydrogenase Activity Colorimetric Assay Kit; Abcam, Cambridge, UK). Positive controls (PC) and NADH standard curves were included. All samples were pipetted into 96-well plates in duplicate. Measurements were performed at 450 nm using a multiplate reader (Tristar 3, Berthold Technologies GmbH & Co. KG, Bad-Wildbad, Deutschland) and recorded every 60 s for 1 h at 37 °C.

##### Nile Red Staining and Lipid/Nuclei-Ratio

On day 14, differentiated cells in 24-well plates were stained with Nile red in order to verify the existence of intracellular lipids. Nuclei were stained with DAPI (staining protocol see Supplementary Table S1). A Leica DMI 6000B Epi-fluorescence microscope (Leica Microsystems GmbH, Wetzlar, Germany) was used for visualization at excitation and emission wavelengths of 480/530 nm (neutral lipids) and 515/590 nm (total lipids), respectively, and for DAPI at 360/470 nm (nuclei). To normalize lipid content for cell density, the lipid/nuclei index was calculated according to Becker et al. [62] after measuring the fluorescence of Nile red (lipid-index) and DAPI (nuclei-index) at 475/570 nm and 358/461 nm, respectively, using the 2300 EnSpire^TM^ multiplate reader (PerkinElmer Inc., Waltham, MA, USA). Measurements were carried out in triplicates.

#### 2.3.2. Osteogenic Differentiation

For osteogenic differentiation, cells were seeded in 25 cm^2^ cell culture flasks and 24-well cell culture test plates (20,000 cells/cm^2^) in duplicate. At 70–90% confluence (day 0), osteogenic induction and differentiation were promoted using an osteogenic differentiation medium up to day 14 or 21. The medium consisted of DMEM with 4.5 g/L glucose (Life Technologies GmbH, Karlsruhe, Deutschland), 10% FBS (Sigma Aldrich, Taufkirchen, Germany), 100 U/mL and 100 µg/mL penicillin-streptomycin (Sigma Aldrich, Taufkirchen, Germany), 2.5 µg/mL amphotericin B (BioWest, Riverside, CA, USA), 0.1 µM dexamethasone (Sigma-Aldrich, Taufkirchen, Germany), 10 mM ß-glycerophosphate and 50 µM ascorbic acid (all from Sigma Aldrich, Taufkirchen, Germany).

##### Alkaline Phosphatase Activity Measurement

On day 14, osteogenic differentiation of cells incubated in 25 cm^2^ cell culture flasks were determined quantitatively by measuring the alkaline phosphatase (ALP) enzyme activity using a fluorometric Alkaline Phosphatase Assay Kit (Abcam, Cambridge, UK). In total, 4 × 10^5^ cells were centrifugated at 300× *g* at 4 °C for 5 min, washed with DPBS with Ca^2+^/Mg^2+^ (Sigma-Aldrich, Taufkirchen, Germany) and centrifuged again (300× *g*, 4 °C, 5 min). The cell pellet was dissolved in 400 µL ALP Assay Buffer. After centrifugation at 13,000× *g* at 4 °C for 3 min, supernatant was removed and the cell pellet was processed according to the ALP assay kit protocol. Measurements of the fluorescence intensities of the samples, which were pipetted in duplicate into 96-well plates, were performed using a 2300 EnSpire^TM^ multiplate reader at excitation and emission wavelengths of 360 nm and 440 nm, respectively.

##### Von Kossa Staining and Index of Osteogenic Differentiation

The osteogenically differentiated cells in 24-well plates were stained according to Von Kossa on day 21 (staining protocol see Supplementary Table S2). Visualization was performed by transmitted light microscopy (AE2000, Motic Deutschland GmbH, Wetzlar, Germany). The optical densities (OD) of the induced (ind) and non-induced NC were measured at a wavelength of 492 nm by means of a 2300 EnSpire^TM^ multiplate reader. Thereafter, the index of osteogenic differentiation (IOD) was determined as the ratio of OD_492nm_ of induced cells and NC [IOD = OD_492nm_ (ind)/OD_492nm_ (NC)]. Measurements were performed in triplicates.

#### 2.3.3. Chondrogenic Differentiation

Chondrogenic differentiation was performed in pellet culture in duplicate using 5 × 10^5^ cells in a 15 mL conical centrifugation tube (TPP Techno Plastic Products AG, Trasadingen, Switzerland). A total of 2 mL chondrogenic differentiation medium was added to the cells, which consisted of DMEM (Life Technologies GmbH, Karlsruhe, Germany), supplemented with 25 mM glucose, 100 U/mL and 100 µg/mL penicillin-streptomycin (Sigma Aldrich, Taufkirchen, Germany), 2.5 µg/mL amphotericin B (BioWest, Riverside, CA, USA), 10 ng/mL TGF-β3 (Life Technologies GmbH, Karlsruhe, Germany), 1% Corning^TM^ ITS + Premix Universal (Fisher Scientific GmbH, Schwerte, Germany), 100 nM dexamethasone, 100 µM ascorbic acid and 400 µM proline, (all from Sigma-Aldrich, Taufkirchen, Germany). Cell pellets were formed by centrifugation at 280× *g* at 4 °C for 5 min. After a 21 d induction period, cell pellets were fixed with ROTI^®^Histofix 4% (Carl Roth GmbH + Co. KG, Karlsruhe, Germany) overnight, embedded in paraffin in the embedding station Logos One (A. Menarini Diagnostics, Berlin, Germany), cut to a layer thickness of 0.5 µm in a rotary microtome (HM325; Thermofisher Scientific, Waltham, MA, USA) and placed onto coated slides (StarFrost Advanced Adhesive; Engelbrecht GmbH, Edermünde (Besse), Germany). NC were cultured in 24-well plates (20,000 cells/cm^2^) in monolayers in duplicate after preliminary experiments (not published) had shown that non-induced cell pellets disaggregate over the 21 d cultivation period and were fixed in ROTI^®^Histofix 4% for further staining procedures.

##### Hematoxylin–Eosin and Alcian Blue Staining

After deparaffinization and rehydration, samples were stained with hematoxylin (Carl Roth GmbH + Co. KG, Karlsruhe, Germany) and eosin (Waldeck GmbH & Co. KG, Münster, Germany) (HE) and Alcian blue in duplicate. Alcian blue was to detect acidic glycosaminoglycans and followed the protocol of the Alcian Blue-Nuclear Fast Red for Acidic Mucosubstances staining kit (Morphisto GmbH, Offenbach am Main, Germany; staining protocols see Supplementary Tables S3 and S4). Similar to the “Bern Score” developed by Grogan et al., which evaluates the safranin O-fast green-stained pellet cultures [63], a scoring system for the Alcian blue-stained pellet cultures was established to classify the chondrogenic differentiation. The criteria were the same as stated in the above-mentioned study [63]. Blinded evaluation was performed by two observers using transmitted light microscopy.

### 2.4. Cardiomyogenic Differentiation

**Figure 1:**
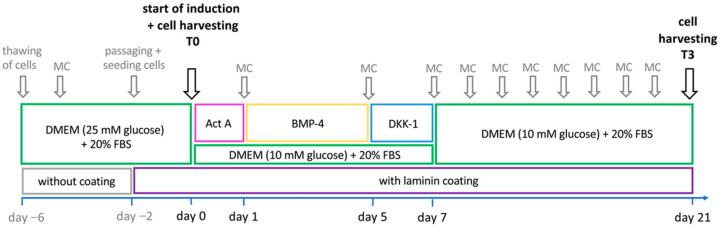
Study design of the cardiomyogenic differentiation experiments using the induction factors activin A (Act A), bone morphogenetic protein-4 (BMP-4) and Dickkopf-1 (DKK-1). In both sub-experiments, equine adipose-derived stem cells were induced with Act A on day 0, with BMP-4 from day 1 to 5 and with DKK-1 from day 5 to 7 in laminin-coated cell culture flasks. Further cultivation from day 7 on was performed in a modified basal medium, and, on day 21, cells were harvested for subsequent molecular–biological studies. Abbreviations: DMEM, Dulbecco’s Modified Eagle’s Medium; FBS, fetal bovine serum; MC, medium change; T0, time point day 0; T3, time point 3 weeks after induction.

Based on our previous study, in which no cardiomyogenic differentiation was achieved using 5-AZA in uncoated cell culture flasks [59], the cardiomyogenic differentiation protocol of the present study applied Act A (Peprotech, Hamburg, Germany) for 24 h, BMP-4 (R&D Systems, Inc., Minneapolis, MN, USA) for 96 h and DKK-1 (Peprotech, Hamburg, Germany) in abd-ASCs-_SVF_ (P3–6) for 48 h (*n* = 5), factors that are involved in embryonic cardiomyogenesis [52,64,65,66,67,68]. In addition, the 25 cm^2^ cell culture flasks were coated with 2 µg/cm^2^ murine laminin (Sigma Aldrich, Taufkirchen, Germany) in order to prevent cell detachment. The study design is shown in Figure 1. The experiment was carried out in two parts in two replicates each in which different concentrations of the induction factors were tested (first sub-experiment: protocols A and B; second sub-experiment: protocols A, B and C). In the first sub-experiment, the cardiomyogenic induction of the 80–90% confluent ASCs was performed (A) with 100 ng/mL Act A, 10 ng/mL BMP-4 and 100 ng/mL DKK-1 and (B) with 100 ng/mL Act A, 20 ng/mL BMP-4 and 100 ng/mL DKK-1. In the second sub-experiment, cardiomyogenic induction protocols included (A) 0 ng/mL Act A, 50 ng/mL BMP-4 and 150 ng/mL DKK-1, (B) 100 ng/mL Act A, 50 ng/mL BMP-4 and 150 ng/mL DKK-1, and (C) 100 ng/mL Act A, 50 ng/mL BMP-4 and 0 ng/mL DKK-1. After seven days of induction, cells were further incubated in a modified basal medium (MB-M) until day 21. MB-M consisted of DMEM supplemented with 20% FBS, 10 mM glucose, 100 U/mL and 100 µg/mL penicillin-streptomycin, 2.5 µg/mL amphotericin B and 15 mM HEPES. On day 0 (T0) and three weeks thereafter (T3), cells were harvested. Gene expression analysis of the cardiac markers *NKX2*-*5*, *GATA4*, *MYH6*, *MYH7* and *TNNI3*, the muscle marker *MYF6* and the pluripotency associated markers *OCT4/POUF5*, *MYC* and *DNMT3B* was performed using SYBR Green reverse-transcription quantitative PCR (RT-qPCR). RT-qPCR analyses were performed according to our previous study in triplicates [59]. The primers used for RT-qPCR are listed in the Supplementary Table S5.

### 2.5. Statistical Analysis

Data analysis was performed using IBM SPSS software, Version 27 and Graph Pad Prism, Version 9.1.2. for Mac, Graph Pad Software. Initially, a descriptive data analysis and a test for normal distribution was carried out (Kolmogorov–Smirnov and Shapiro–Wilk tests, visual examination of the histograms, QQ-plots and boxplots). A significance level of 5% was set for all experiments (α = 0.05). Repeated measures analysis of variance (ANOVA) was performed to examine cell growth and PDT with cell numbers and PDT of consecutive days (representing the repeated measurements). A Greenhouse–Geisser correction was used. Non-parametric tests were used in the trilineage differentiation experiments to assess the effect of tissue localization among abd-ASCs-_EXP_, rb-ASCs-_EXP_ and sc-ASCs-_EXP_ (Kruskal–Wallis test) and the effect of isolation techniques between abd-ASCs-_EXP_ and abd-ASCs-_SVF_ (Mann–Whitney U test). When data were collected for both induced cells and NC, treatment effects were additionally examined using the Mann–Whitney U test. In case of multiple comparisons, significance values were adjusted with the Bonferroni correction [69]. Data are shown in dot plots with medians and interquartile ranges (IQR). In order to evaluate the cardiomyogenic differentiation potential, a univariate ANOVA was carried out, as well as a Tukey HSD post-hoc test. Here, dot plots show mean values ± standard deviations (SD). In accordance with previous experiments, statistical tests of RT-qPCR were performed only if C_t_ mean values of individual markers and treatments were more than four cycles away from the C_t_ mean values of non-RT and H_2_O technical control samples [59]. Experiments were performed without replicates if not stated otherwise.

## 3. Results

### 3.1. Proliferation Assay

**Figure 2:**
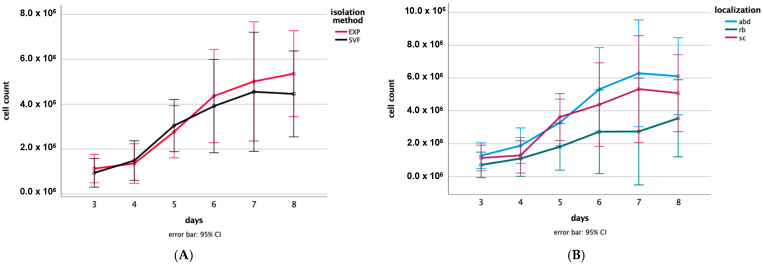
Cell growth curves of equine adipose-derived stem cells of passage 3. The proliferation ability of ASCs-_SVF_ (black) und ASCs-_EXP_ (red), which were isolated from abdominal (abd, blue), retrobulbar (rb, green) and subcutaneous (sc, dark red) adipose tissue of *n* = 3 horses, were determined on days 3 to 8. Cell counts are summarized for isolation techniques (**A**) and adipose tissue localizations (**B**).

A high proliferation potential was demonstrated in cells of all tested origins and isolation methods. After seeding, cells quickly adapted to the culture conditions (~2 days). An exponential cell growth followed until days 5–6. Cell growth curves are shown in Figure 2.

Significant differences were observed in cell counts among days (*p* < 0.001) but not among isolation techniques (*p* = 0.64) and localizations (*p* = 0.35), using repeated measures ANOVA; although, subjectively, abd-ASCs-_EXP_ showed the best growth. Regarding days, significant differences were detected between days 4 and 5 (*p* < 0.001), days 5 and 6 (*p* = 0.007), as well as days 6 and 7 (*p* = 0.049) by means of the Greenhouse–Geisser test. Interactions between days and isolation techniques (*p* = 0.64) as well as the days and localizations (*p* = 0.35) were not significant, i.e., the influence of the localizations and isolation techniques were the same at different time points. Additionally, with regard to the PDT, neither a significant localization effect (*p* = 0.48) nor a significant isolation technique effect (*p* = 0.91) was shown, i.e., the PDT of different adipose tissues and isolation methods did not differ significantly. The cell counts and PDTs of individual groups can be found in Supplementary Table S6.

### 3.2. Trilineage Differentiation Potential

#### 3.2.1. Adipogenic Differentiation

Adipogenic differentiation was observed in all samples by transmitted light microscopy, except for rb-ASCs-_EXP_ of one horse. While the NC retained their fibroblast-like cell morphology, the adipogenically induced cells changed their morphology to large rhomboid and drop-shaped cells containing lipid droplets from day five on.

##### Glycerol-3-Phosphate Dehydrogenase Assay

**Figure 3:**
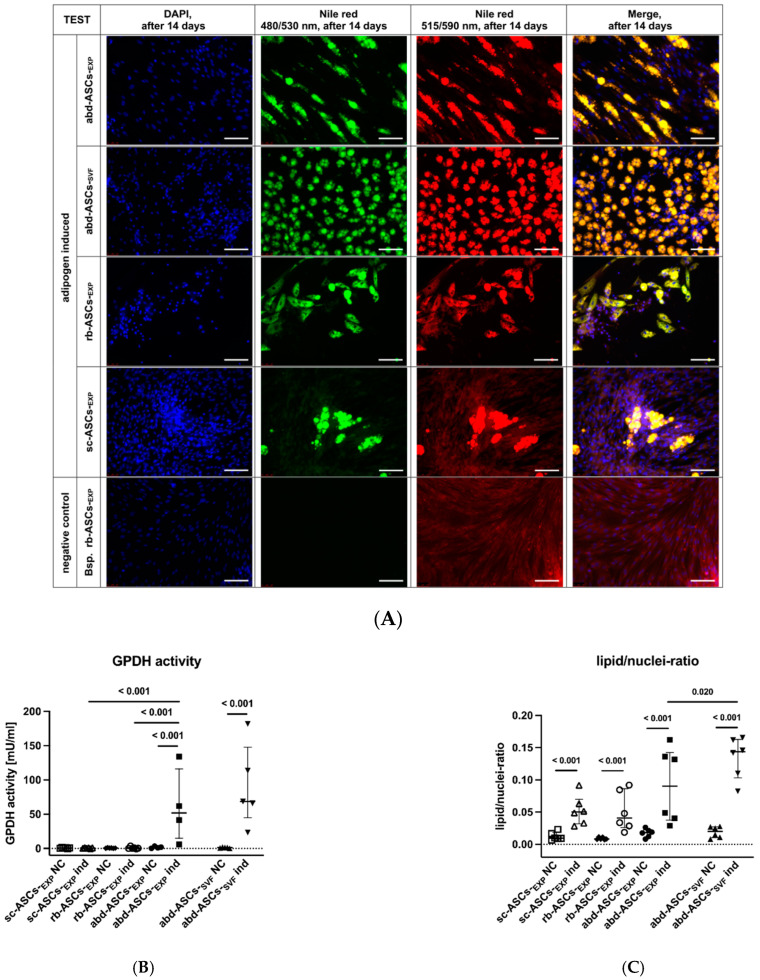
Adipogenic differentiation. (**A**) Fluorescence microscopic images of adipogenically induced equine adipose-derived stem cells (ASCs) after Nile red and DAPI staining of one representative horse on day 14. Preadipocyte differentiation was induced in ASCs harvested from abdominal (abd), retrobulbar (rb) and subcutaneous (sc) adipose tissue using the explant method (EXP) or using the stroma vascular fraction (SVF) after collagenase digestion. Cell nuclei were stained blue (DAPI), non-polar lipids green and total lipids red (Nile red). In all induced samples, except for non-induced negative control (NC), lipid droplets were detected. Scale bar: 100 µm (20× objective). (**B**) Glycerol-3-phosphate dehydrogenase (GPDH) activity and (**C**) Lipid/nuclei-ratio of adipogenically induced equine ASCs measured on day 14. NC were included in the experiment. Median values are shown with interquartile ranges as error bars (*n* = 6). The *p*-values shown are from the Mann–Whitney U test (comparisons of isolation methods and treatment effects) or from the Kruskal–Wallis test (comparisons of adipose tissue localizations).

Upon examination of the GPDH activity, a significant localization effect was demonstrated (Kruskal–Wallis test: *p* < 0.001). GPDH activity was higher in abd-ASCs-_EXP_ compared to rb-ASCs-_EXP_ (*p* < 0.001) and sc-ASCs-_EXP_ (*p* < 0.001), with no difference between the latter two (*p* > 0.99). No significant difference was detected between induced abd-ASCs-_EXP_ and abd-ASCs-_SVF_ (Mann–Whitney U Test: *p* = 0.28). When comparing the GPDH activity of induced cells and NC, a significant treatment effect was demonstrated in abd-ASCs-_EXP_ and abd-ASCs-_SVF_ (Mann–Whitney U Test: both *p* < 0.001). No GPDH activity (except for rb-ASCs-_EXP_ of one horse) and therefore no significant treatment effects were detected in rb-ASCs-_EXP_ (Mann–Whitney U Test: *p* = 0.18) and sc-ASCs-_EXP_ (Mann–Whitney U Test: *p* = 0.51). GPDH activities of induced and non-induced cells are shown in Figure 3B.

##### Nile Red Staining and Lipid/Nuclei-Ratio

In line with the transmitted light microscopic observations, fluorescence microscopy after Nile red staining verified that all samples exhibited lipid droplet formation (apart from rb-ASCs-_EXP_ of one horse) with, subjectively, the highest formation in abd-ASCs-_SVF_. In Figure 3A, fluorescence microscopic images after Nile red and DAPI staining are shown exemplarily for one horse. The lipid/nuclei-ratio was determined 14 days after induction. No significant differences were detected between ASCs-_EXP_ of abd, rb and sc adipose tissues (Kruskal–Wallis test: *p* = 0.078). When comparing abd-ASCs-_EXP_ and abd-ASCs-_SVF_, on the other hand, a significant difference was detected (Mann–Whitney U Test: *p* = 0.020), i.e., induced abd-ASCs-_SVF_ had a significantly higher lipid/nuclei-ratio compared to induced abd-ASCs-_EXP_ (Figure 3C). All treatment effects proved significant (Mann–Whitney U Test: all *p* < 0.001), i.e., all induced cells showed a significantly higher lipid/nuclei-ratio compared to their corresponding non-induced NC.

#### 3.2.2. Osteogenic Differentiation

**Figure 4:**
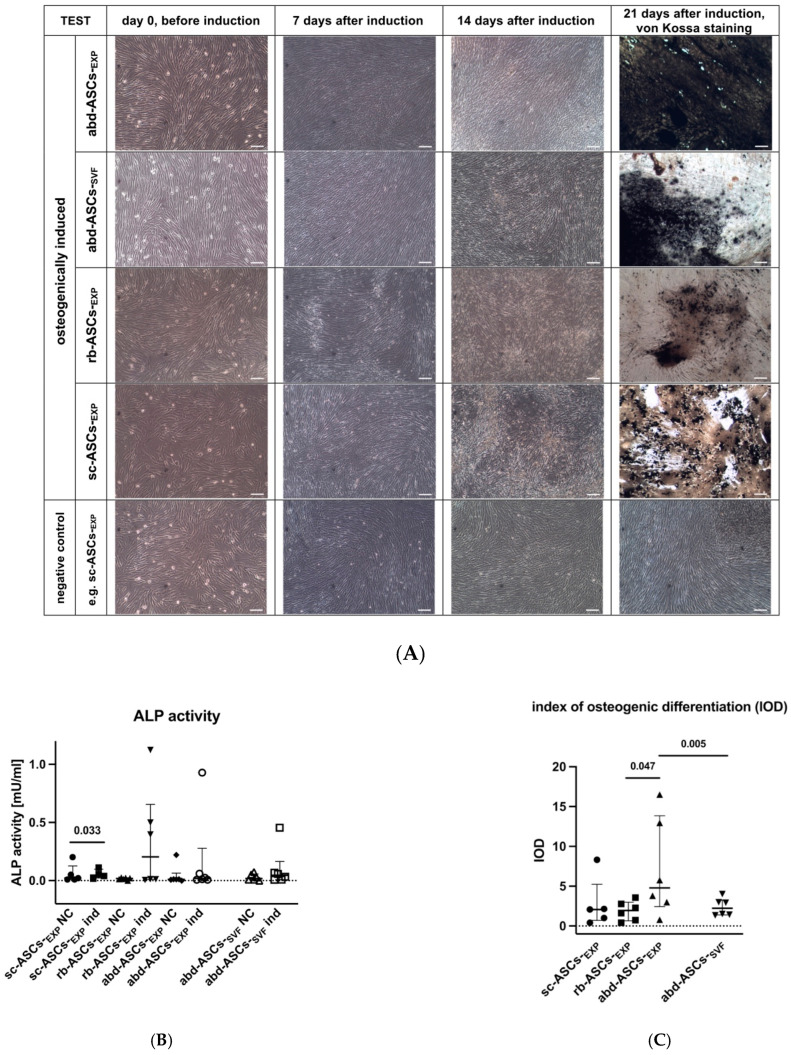
Osteogenic differentiation. (**A**) Transmitted light microscopic images of the osteogenic differentiation of equine adipose-derived stem cells (ASCs) of one representative horse. ASCs were generated from abdominal (abd), retrobulbar (rb) and subcutaneous (sc) adipose tissue using the explant method (EXP) or using the stroma vascular fraction (SVF) after collagenase digestion. Images were taken either before (day 0) and after induction (day 7, 14 and 21). Two weeks after induction, induced cells showed bright shining, punctual, extracellular deposits compared to non-induced negative controls (NC), which increasingly turned into blackish deposits. On day 21, the calcium phosphate deposits were detected by means of Von Kossa staining. Scale bar: 100 µm (10× objective). (**B**) Alkaline phosphatase activity (on day 14) and (**C**) indices of osteogenic differentiation (IOD) of osteogenically induced equine ASCs (on day 21) are shown for induced (ind) and NC cells. The median value and interquartile range (error bar) are shown (*n* = 6). The *p*-values shown are from the Mann–Whitney U test (comparisons of isolation methods and treatment effects) or from the Kruskal–Wallis test (comparisons of adipose tissue localizations).

Osteogenic differentiation was observed in all samples by transmitted light microscopy with the first punctual, bright-shining deposits appearing in the extracellular matrix (ECM) from day 11 onwards. These were transformed into blackish, punctual to extensive deposits over the later course of induction. The extracellular calcium phosphate deposits were verified by Von Kossa staining on day 21. Subjectively, abd-ASCs-_EXP_ showed the highest osteogenic differentiation potential, and no ECM-deposits could be observed within all NC (Figure 4A).

##### Alkaline Phosphatase Activity Measurement

No significant localization effect was detected when comparing induced abd-ASCs-_EXP_, rb-ASCs-_EXP_ and sc-ASCs-_EXP_ (Kruskal–Wallis Test: *p* = 0.06; Figure 4B). When comparing abd-ASCs-_EXP_ and abd-ASCs-_SVF_, no significant effect of isolation technique was detected (Mann–Whitney U Test: *p* = 0.38). Regarding treatment effects, alkaline phosphatase (ALP) appeared slightly higher in all induced samples (except for one horse); however, a significantly increased enzyme activity of ALP was detected only for sc-ASCs-_EXP_ ind vs. NC (Mann–Whitney-U-Test: *p* = 0.033) but not for rb-ASCs-_EXP_ (*p* = 0.41), abd-ASCs-_EXP_ (*p* = 0.21) and abd-ASCs-_SVF_ (*p* = 0.18).

##### Index of Osteogenic Differentiation

After Von Kossa staining, calcium phosphate deposits were semi-quantified by the IOD on day 21 (Figure 4C). A significant localization effect was detected (Kruskal–Wallis Test: *p* = 0.049) with significantly higher IOD in abd-ASCs-_EXP_ compared to rb-ASCs-_EXP_ (*p* = 0.047); the other adipose tissue localizations did not differ significantly from each other (abd-ASCs-_EXP_ vs. sc-ASCs-_EXP_: *p* = 0.33; rb-ASCs-_EXP_ vs. sc-ASCs-_EXP_: >0.99). Furthermore, abd-ASCs-_EXP_ showed a significantly higher IOD than abd-ASCs-_SVF_ (Mann–Whitney U Test: *p* = 0.005).

#### 3.2.3. Chondrogenic Differentiation

**Figure 5:**
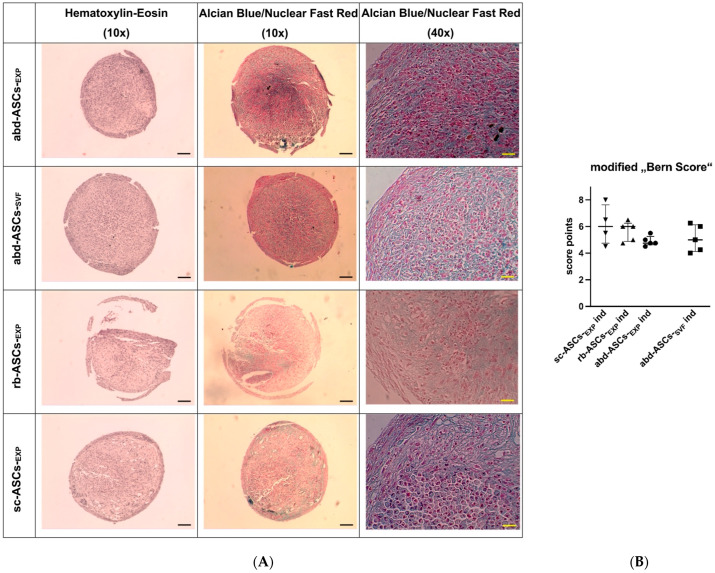
Chondrogenic differentiation. (**A**) Transmitted light microscopic images of chondrogenically induced adipose-derived stem cells (ASCs) of one representative horse. ASCs were generated from abdominal (abd), retrobulbar (rb) and subcutaneous (sc) adipose tissue using the explant method (EXP) or using the stroma vascular fraction (SVF) after collagenase digestion. Cells were subsequently stained with hematoxylin–eosin and Alcian Blue-Nuclear Fast Red to detect mucopolysaccharides on day 21. Scale bar (black): 100 µm (10× objective), scale bar (yellow): 25 µm (40× objective). (**B**) The chondrogenic differentiation potential of equine ASCs was assessed using the modified “Bern Score” according to [63]. Due to the small size of the cell pellets, the preparation of histological sections was not possible for all samples. The dot plot shows median values and interquartile ranges (error bars) (*n* = 4 to 5).

Chondrogenesis was observed by transmitted light microscopy subjectively in all samples. Initially, an overview staining was achieved with HE. Alcian blue stains acidic mucopolysaccharides produced by chondroblasts in the cartilage matrix in blue, whereas nuclear fast red stains cell nuclei red (Figure 5A). In all samples, an accumulation of acidic mucopolysaccharides was detected (abd-ASCs-_EXP_, abd-ASCs-_SVF_, rb-ASCs-_EXP_ and sc-ASCs-_SVF_), with no subjective difference among groups. In accordance with the observations made by transmitted light microscopy, no significant differences were detected between the two isolation methods (Mann–Whitney U Test: *p* = 0.66) and adipose tissue localizations (Kruskal–Wallis Test: *p* = 0.15) using the modified “Bern Score” (Figure 5B), i.e., adipose tissue localizations and isolation methods generated similar chondrogenic differentiation potential.

### 3.3. Cardiomyogenic Differentiation

**Figure 6:**
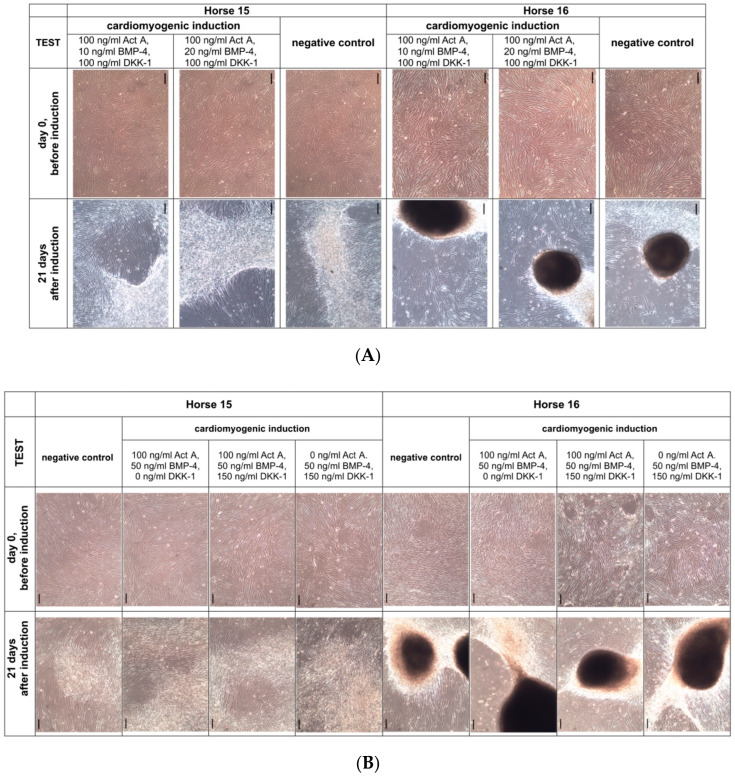
Application of substances for cardiomyogenic differentiation. Cell morphology of equine adipose-derived stem cells exposed to different concentrations of activin (Act) A, bone morphogenetic protein (BMP)-4 and Dickkopf (DKK)-1 in (**A**) a first and (**B**) a second sub-experiment. Cell morphology of two representative horses is shown before (day 0) and 21 days after cardiomyogenic induction. Neither relevant differences between the induced cells and non-induced negative controls nor a cardiomyogenic differentiation could be detected. Cells retained their fibroblast-like cell morphology and formed “domes”, which were more obvious in horse 16 compared to horse 15, particularly three weeks after induction. Scale bar: 100 µm (10× objective).

After consecutive incubation with Act A for 24 h, BMP-4 for 96 h and DKK-1 for 48 h, no changes of cell morphology towards a cardiomyogenic phenotype and no spontaneous beating activity of cells were detected. This applied to all concentrations of the induction factors. Cells retained their fibroblast-like morphology, and “dome” formation was observed due to over-confluent cell growth, which was of different intensity in the five donor horses (see Figure 6A for the first and Figure 6B for the second sub-experiment). These cell morphological observations were also confirmed by SYBR Green RT-qPCR. Neither an upregulation of the cardiac markers *MYH6*, *MYH7*, *TNNI3* nor of the early cardiac markers *NKX2-5* und *GATA4* were observed in both sub-experiments. However, significant downregulation of the pluripotency-associated markers *MYC*, *OCT4/POUF5* and *DNMT3B* was observed from time point T0 until three weeks after induction. All RT-qPCR-results are shown in detail in Supplementary Figures S1 and S2.

## 4. Discussion

The present study aimed to examine and compare the proliferation and differentiation potential of equine ASCs isolated from different adipose tissues via two isolation methods. The main findings of our study were that isolation of equine ASCs from abd, rb and sc adipose tissue is possible using the EXP and SVF isolation methods and that these ASCs showed a high proliferation and adipogenic, osteogenic and chondrogenic differentiation potential. However, under the test conditions selected, a cardiomyogenic differentiation of equine ASCs could not be achieved with Act A, BMP-4 and DKK-1.

This study is one of the first to compare equine ASCs from different adipose tissues. In the literature, there are some studies in horses in which retroperitoneal, inguinal, subcutaneous adipose tissue, lipoma tissue or adipose tissue from the mesentery of the small colon were compared on the basis of their differentiation and proliferation potential [17,27,70]. There have been no studies on horses that examined ASCs from rb adipose tissue until today. Tissue source may have a direct impact on the in vitro properties of derived MSCs [14,27]—even the cut depth during tissue sampling showed such influence [71,72,73]. The study of Baglioni et al. demonstrated that deep-lying visceral adipose tissues have a lower proliferation and differentiation potential compared to sc adipose tissue [71]. In the present study, it was first hypothesized that deep-lying rb adipose tissue has a lower potential in this regard. On the contrary, we showed in the experiments that equine ASCs had a comparably high proliferation potential, regardless of the adipose tissue source. We used a DMEM high glucose medium to culture the equine ASCs from different sources. In other species, it was described that decreasing glucose concentration increases the proliferation ability of MSCs [74,75]. It could thus be speculated that an even better proliferation could have been achieved by using a lower glucose concentration in the present study. However, PDT in the present study amounted to an average of about 41–58 h, which is in rather good agreement with literature data, where PDTs of 40–46 h [60] or 2,2 days (=approx. 53 h) have been reported for horses [76]. It has also been described in previous studies for horses [14,77] and rats [78] that ASCs have a higher proliferation potential than MSCs from umbilical cord blood (UCB-MSCs) and bone marrow (BM-MSCs). The growth potential of MSCs has not only a high relevance for the establishment of in vitro models, but also a high clinical relevance for regenerative medicine [13,14,79].

By examining the trilineage differentiation potential in vitro, conclusions can be drawn on the multipotent characteristics of MSCs. Consequently, a multipotent differentiation potential could be demonstrated in abd-ASCs-_EXP_, abd-ASCs-_SVF_, rb-ASCs-_EXP_ and sc-ASCs-_EXP_ in this study, whereby differences could be found in cells of different isolation methods and adipose tissue localizations. It had already been proven in various studies that the tissue origin of MSCs plays a decisive role [14,15,17,27]. In adipogenic differentiation, we showed that the use of 10% FBS led to a lipid droplet accumulation that was observed from the fifth day on. Other studies selected considerably less induction time (e.g., three days) and the use of rabbit serum [14,15,29]. According to various studies, a Nile red staining [31,61,62] and measurement of the lipid/nuclei-ratio [61,62] were performed. Contrary to studies of Lee et al. and Gittel et al. [19,29], ASCs-_SVF_ exhibited a significant higher adipogenic differentiation potential compared to ASCs-_EXP_ on day 14, but only abd-ASCs were examined in the present study. In addition, significant localization effects were shown within the GPDH activity measurement. Even if GPDH activity measurement has not yet been implemented in horses’ literature as standard procedure, GPDH represents an important differentiation marker, as described in various studies for human [80,81] and murine MSCs [82]. It can be assumed that increased GPDH activity may be beneficial for cellular utility in cell-based therapy. In cattle, for example, Becker et al. suggested that increased GPDH activity could help sequester excessive serum lipids, which may prevent lipid accumulation in other tissues. Moreover, adipogenically induced bovine ASCs demonstrated highest GPDH activity at the lowest insulin concentration [62]. Hence, we suggest that horses with hyperinsulinemia, as in equine metabolic syndrome (EMS), might have a lower GPDH activity. This is in accordance with Marycz et al., who demonstrated that equine EMS-ASCs have a lower adipogenic differentiation capacity [83]. Therefore, ASCs for cell-based therapies should not be derived from EMS-affected donors. Regarding adipogenic differentiation in horses, significant localization effects were also described by Arnhold et al., who demonstrated that ASCs from retroperitoneal adipose tissue had a higher adipogenic differentiation potential than ASCs from lipoma and sc adipose tissue [27]. On the contrary, it was proven in rats that sc-ASCs exhibited a higher adipogenic differentiation potential than visceral ASCs because of a lower expression of stemness markers amongst others [84]. However, how far these markers of stemness differ in equine abd-ASCs compared to rb- and sc-ASCs was not investigated in the present study. Finally, a number of studies observed no significant localization effects regarding adipogenic differentiation potential of equine [76,85] and rabbit MSCs [86].

In various studies, MSCs of different species and tissue sources, consistent with our work, have been shown to have a high osteogenic differentiation potential [15,17,27,60,76]. MSCs from umbilical cord blood and tissue have a lower osteogenic differentiation potential than ASCs, BM-MSCs and MSCs from tendon tissue (TT-MSCs) [14]. Equine ASCs have a particularly high potential for in vitro osteogenesis compared to human [15], porcine and canine ASCs [87]. In the present study, osteogenesis was demonstrated by means of Van Kossa staining and determination of the IOD on day 21 [14,15,27,29,88]. In addition, an ALP assay was performed on day 14 in accordance with other studies [27,89,90]. Samples from all but one horse showed slightly increased and sc-ASCs-_EXP_ significantly increased ALP activity. ALP represents an important marker within osteoblast differentiation [89]. Additionally, on day 14, a matrix mineralization was observed by transmitted light microscopy, which was, compared to the ALP activity, least pronounced in the one horse. In the study by Gittel et al., no significant differences were shown between ASCs of EXP and SVF isolation methods when measuring the IOD, but only sc-ASCs were examined in their study [29]. In the present work, on the other hand, abd-ASCs-_EXP_ exhibited a significantly higher IOD compared to abd-ASCs-_SVF_, as well as abd-ASCs-_EXP_ compared to rb-ASCs-_EXP_.

Chondrogenic differentiation was performed in a standard 3D micromass pellet culture according to previous studies [14,15,91,92]. NC in pellet culture could not be included because preliminary tests had shown that a compact pellet structure could not be achieved without using chondrogenic induction factors and that the pellet could even disintegrate, as also described in previous studies [93,94]. A chondrogenic differentiation potential could be detected in all samples after Alcian blue staining, where no differences could be detected between ASCs from the different localizations and isolation methods in the present study using the modified “Bern Score” [63]. On the other hand, significant differences between ASCs of different adipose tissue localizations could be demonstrated in the study by Arnhold et al., in which ASCs from retroperitoneal and sc adipose tissue showed a higher chondrogenic differentiation potential than ASCs from lipoma fat [27]. A comparable chondrogenic differentiation potential of MSCs of the SVF and EXP isolation methods was demonstrated in other studies [19,29]. The data suggest that equine ASCs from abd, rb and sc adipose tissue may be suitable for clinical use in cartilage regeneration. However, further investigation is necessary, especially regarding the paracrine activity of the cells. Moreover, studies investigating the immunomodulatory properties of MSCs based on their tissue origin would be advisable, as has already been described for different species [95,96,97,98]. The investigation of the in vitro chondrogenesis represents an important basis for the clinical application of MSCs in equine orthopedics. In horses, the first drug (“Arti-Cell^®^Forte”, Boehringer Ingelheim Vetmedica GmbH, Ingelheim am Rhein, Germany) based on chondrogenically preconditioned MSCs from peripheral blood was approved for the market in 2019 to reduce lameness in aseptic joint diseases [99,100,101]. In the case of adipogenic, osteogenic and chondrogenic differentiation, follow-up studies would be advisable in which not only ASCs-_EXP_ and ASCs-_SVF_ from the abd localization, but also ASCs of the EXP and SVF methods from rb and sc adipose tissue are compared with each other.

The cardiomyogenic differentiation potential was examined solely in equine abd-ASCs-_SVF_ since previous studies in other species usually used ASCs-_SVF_ for in vitro cardiomyogenesis [47,49,102,103]. In the present study, abd-ASCs also achieved good results regarding the proliferation and trilineage differentiation potential. However, there have not yet been studies by other research groups examining the cardiomyogenic differentiation potential in equine ASCs. The success of the cardiomyogenic differentiation depends not only on the cell type chosen (multipotent vs. pluripotent), but also on the differentiation media, the induction factor concentrations and the cell culture model selected (2D vs. 3D) [104,105]. Compared to the induction factor 5-AZA used in our previous study [59], there are comparatively few publications on Act A, BMP-4 and DKK-1 in which these factors were used in the cardiomyogenic differentiation of ASCs. To the best of the authors’ knowledge, the protocol chosen in this study has so far not been used in MSCs. However, there are studies in humans and rodents using either BMP-4 alone [46,48,106] or in combination with other factors, such as bFGF [46], VEGF [47] or 5-AZA [48]. For the cardiomyogenic induction of pluripotent embryonic stem cells (ESCs) and iPSCs, the factors Act A, BMP-4 and DKK-1 are used much more frequently [54,107,108] than in MSCs. In this regard, for example, 10 ng/mL [107] or even 100 ng/mL Act A [54] were used for 24 h, 10 ng/mL BMP-4 for 4 days and DKK-1 on days 5 to 7 [108] or on days 5 to 11 [54], as in the present study. DKK-1 should not be used on days two to three, as it initially inhibits the induction of cardiomyogenesis [50]. Furthermore, Van Dijk et al. hypothesized that when ASCs attach to ECM molecules, e.g., laminin, there is an increase in stem cell differentiation. These ECM molecules are physiologically present in healthy hearts and are upregulated during myocardial infarction. In particular, laminin plays a role in the late cardiomyogenic differentiation of ASCs in vitro [103]. In the present study, however, a cardiomyogenic differentiation of equine abd-ASCs-_SVF_ could not be achieved by the induction factors Act A, BMP-4 and DKK-1 in combination with a laminin coating. An upregulation of cardiac markers in the induced cells compared to the non-induced NC was not detected by means of RT-qPCR. At time point T0, the expression of pluripotency-associated markers *OCT4/POUF5*, *MYC* and *DNMT3B* were confirmed in the cells examined. Within all induction experiments, a significant downregulation of the gene expression of these markers was observed at time point T3, both in induced and non-induced cells (apart from *DNMT3B* and *OCT4/POUF5*, T0 vs. T3-NC, in the second sub-experiment). Studies in rodents and humans have shown that when MSCs are induced to differentiate, there is a downregulation of pluripotency-associated ESC markers [109,110]. In the present study, however, not only induced cells were affected by this downregulation, but also NC, so that cardiomyogenic differentiation activity was excluded. That this downregulation can be associated with the occurrence of senescence in cells has already been discussed in detail in Trachsel et al. [59]. While some studies demonstrated that BMPs possess a cardiomyogenesis-promoting effect [106,111,112], other research groups showed that transient inhibition of BMPs is necessary for in vitro cardiomyogenesis [25,113]. Although many different in vitro differentiation protocols were studied in the literature, it has not yet been possible to find the optimal cardiomyogenic inducer [114], including this study in horses. Our previous study also failed to determine cardiomyogenic induction by using 5-AZA [59], as has already been described in other studies, particularly for human ASCs [110,115]. The need for future studies further investigating in vitro cardiomyogenesis of MSCs is evident [116].

Due to the heterogeneity of the study population (different breed, age, sex and pre-existing conditions of horses), which must be regarded as a limitation, the present study did not focus on the individual animal itself, but rather on the comparison of MSCs from different isolation techniques and adipose tissue localizations. Follow-up studies with a more homogenous donor cell population are advised. It should also be considered that the passage number, tissue source, isolation method and culture conditions can influence the characteristics of cells in vitro, as has already been described in various studies [26,33].

## 5. Conclusions

The results obtained in the present study regarding the proliferation and multilineage differentiation potential of equine ASCs provide important insights for preclinical studies in large animal models and represent a basis for translational medicine (clinical applicability of MSCs), even if the in vivo behavior of the cells certainly cannot be translated one-to-one from the in vitro behavior. Further studies are advisable in which the paracrine mechanisms of MSCs are examined on large animal models. Regarding the cardiomyogenic differentiation potential, it is recommended to use pluripotent stem cells instead of multipotent ASCs, especially iPSCs, which have a higher cardiomyogenic differentiation potential due to their pluripotency behavior. The knowledge gained from in vitro studies on large animal models can also be relevant for questions relating to the application of MSCs in human medicine.

## Data Availability

All data are included in the manuscript and its supplementary material.

## Supplementary Materials

Supplementary information is available online at https://www.mdpi.com/article/10.3390/ani13081352/s1; Supplementary Tables S1–S4: Staining protocols, Supplementary Table S5: List of primers, Supplementary Table S6: Cell counts and population doubling time, Supplementary Figures S1 and S2: Results of SYBR Green RT-qPCR.
