# Supplementary material for: Multilineage Differentiation Potential of Equine Adipose-Derived Stromal/Stem Cells from Different Sources
Source: Animals (Basel). 2023 Apr 15;13(8):1352. doi: 10.3390/ani13081352 (PMC10135324; doi:10.3390/ani13081352)
Supplement: Supplementary file 1 [file animals-13-01352-s001.zip › Supplementary Figure S1-S2_Results of SYBR Green RT-qPCR.pdf]

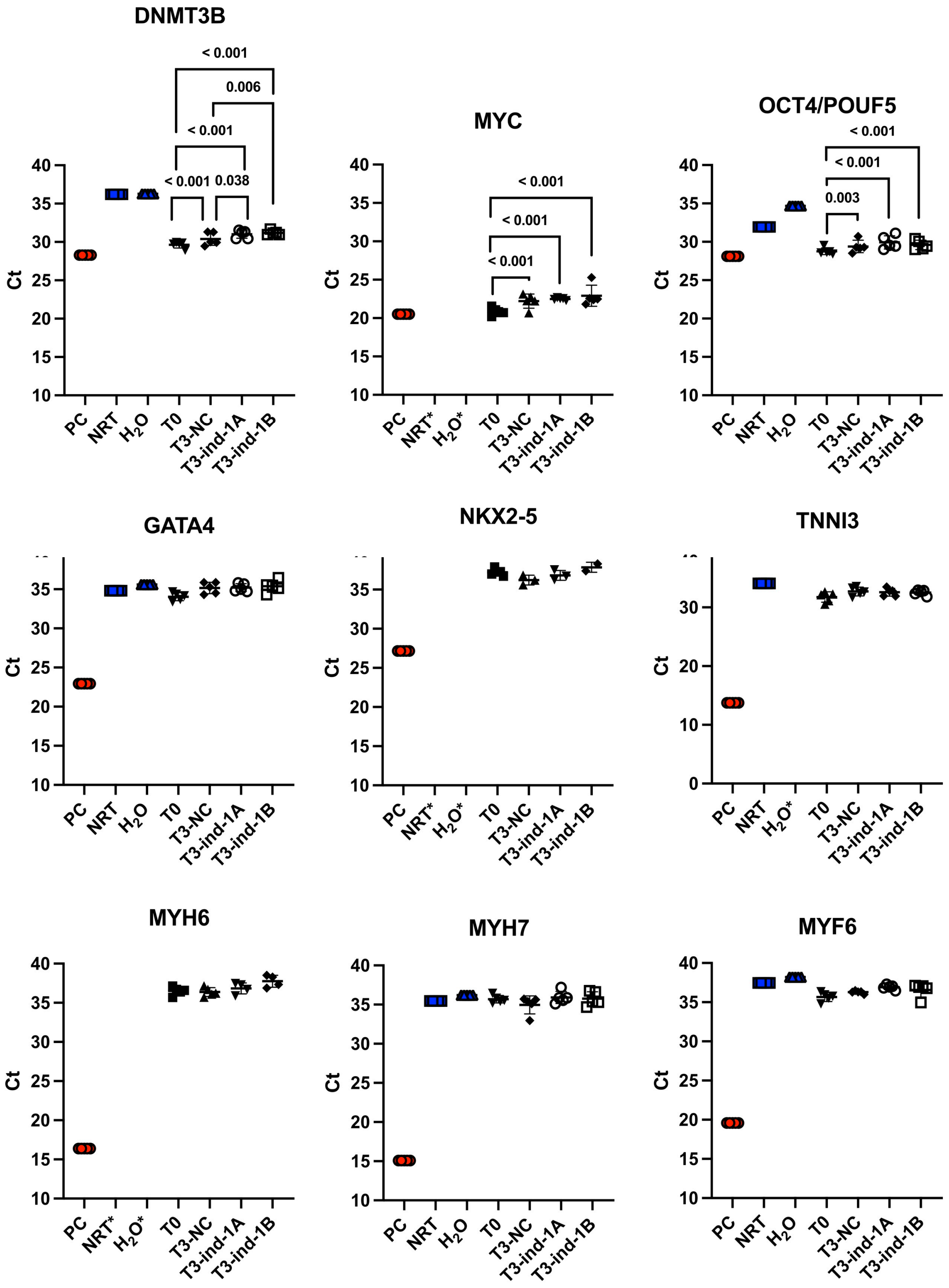

Supplementary Figure S1: Results of SYBR Green RT-qPCR (first sub-experiment) for the cardiac, myogenic and pluripotency-associated markers expressed in ASCs before (T0, non-induced) and three weeks after induction with activin A (Act A), bone morphogenetic protein-4 (BMP-4) and Dickkopf-1 (DKK-1) (T3-ind-1A, induced with 100 ng/ml Act A, 10 ng/ml BMP 4 and 100 ng/ml DKK 1; T3-ind-1B: induced with 100 ng/ml Act A, 20 ng/ml BMP-4 and 100 ng/ml DKK-1; T3-NC, negative control). In addition, positive (p, red) and negative controls (NRT and H_2O) are shown. The trial was performed in two experimental runs (*N* = 5 donors). In the dotplots, the C_t mean values ± standard deviations (error bars) are shown. For the statistical analysis, a one-way ANOVA (post hoc: Tukey´s HSD) was performed and statistical significance was assumed at $p < 0.05$. **Abbreviations:** DNMT3B: DNA methyltransferase 3 beta, GATA4: GATA binding protein 4, ind: induced, MYC: MYC proto-oncogen, MYF6, myogenic factor 6, MYH6/7: myosin heavy chain 6/7, NC: negative control, NKX2-5: NK2 homeobox 5, NRT: no reverse transcriptase, OCT4/POUF5: octamer-binding protein 4/POU class 5 homeobox 1, TNNI3: troponin I3, cardiac type. *C_t mean > 40.

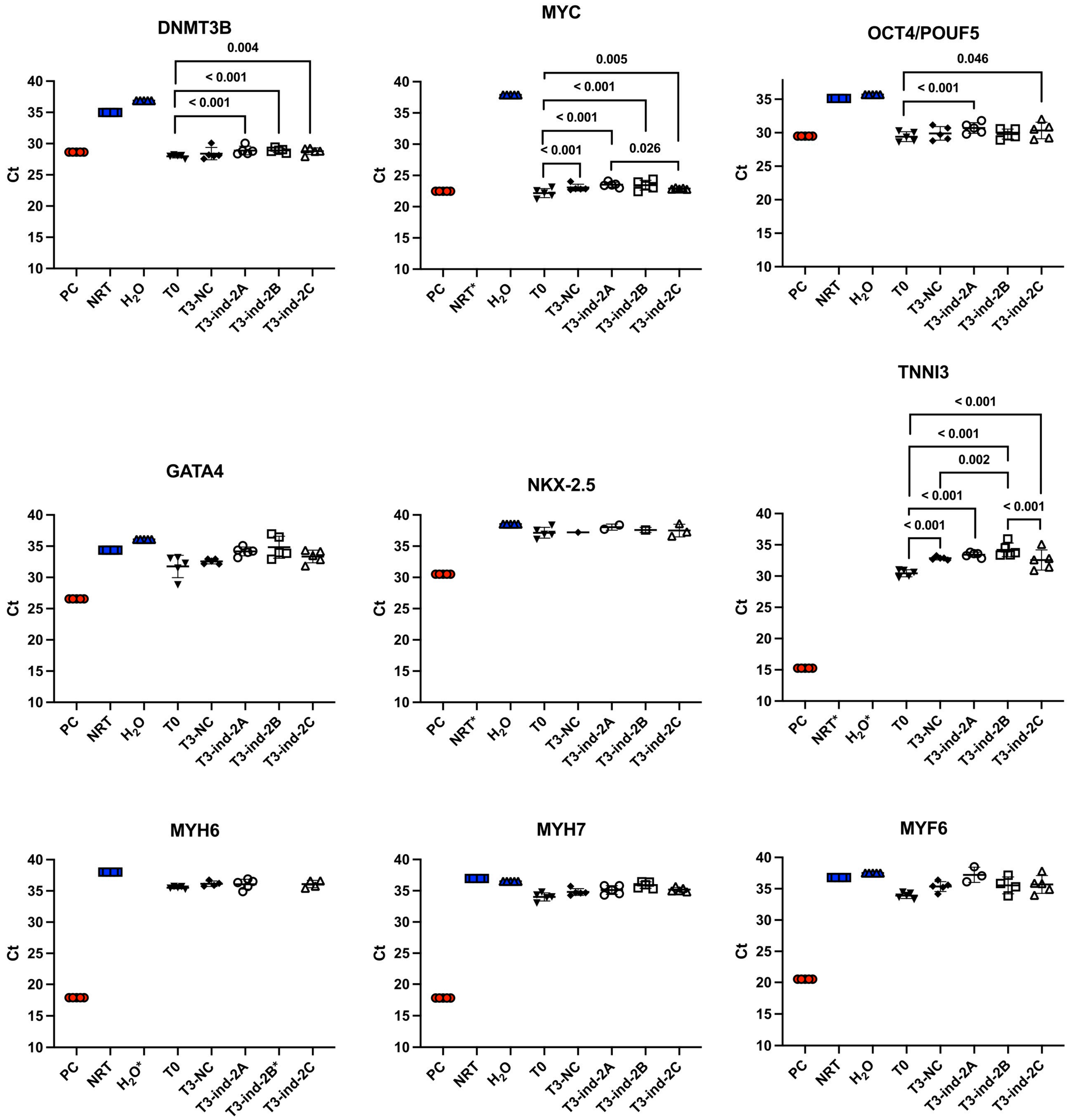

Supplementary Figure S2: Results of SYBR Green RT-qPCR (second sub-experiment) for the cardiac, pluripotency-associated and myogenic markers expressed in ASCs before (T0, non-induced) and three weeks after induction with activin A (Act A), bone morphogenetic protein-4 (BMP-4) and Dickkopf-1 (DKK-1) (T3-ind-2A: induced with 0 ng/ml Act A, 50 ng/ml BMP-4 and 150 ng/ml DKK-1; T3-ind-2B: induced with 100 ng/ml Act A, 50 ng/ml BMP-4 and 150 ng/ml DKK-1; T3-ind-2C: induced with 100 ng/ml Act A, 50 ng/ml BMP-4 and 0 ng/ml DKK-1; T3-NC, negative control). Additionally, positive (p, red) and negative controls (H_2O and NRT) are shown. The trial was conducted in two experimental runs (*N* = 5 donors). The C_t mean values and standard deviations (error bars) are shown in the dotplots. A one-way ANOVA (post hoc: Tukey´s HSD) was performed for the statistical analysis, and statistical significance was assumed at $p < 0.05$. **Abbreviations:** see Supplementary Figure 1; *C_t mean > 40.
