# Supplementary material for: Multilineage Differentiation Potential of Equine Adipose-Derived Stromal/Stem Cells from Different Sources
Source: Animals (Basel). 2023 Apr 15;13(8):1352. doi: 10.3390/ani13081352 (PMC10135324; doi:10.3390/ani13081352)
Supplement: Supplementary file 1 [file animals-13-01352-s001.zip › Supplementary Table S1-S4_Staining protocols.pdf]

Supplementary Table S1–S4 – Staining protocols:

Table S1: Staining protocol Nile red and DAPI (adipogenic differentiation).

Time / cycles	Work step	Chemical
2x	Washing	DPBS with Ca^{2+}/Mg^{2+}
60 min	Fixation	ROTI®Histofix 4 %
2x	Washing	DPBS with Ca^{2+}/Mg^{2+}
5 min, in the dark	Staining of lipids	Nile red (1:100 diluted with DPBS with Ca^{2+}/Mg^{2+})
1x	Washing	DPBS with Ca^{2+}/Mg^{2+}
5 min, in the dark	Permeabilisation	Triton-X-100 (0,5%)
1x	Washing	DPBS with Ca^{2+}/Mg^{2+}
5 min, in the dark	Nuclear staining	DAPI (1:5000 diluted with DPBS with Ca^{2+}/Mg^{2+})
2x	Washing	DPBS with Ca^{2+}/Mg^{2+}

Table S2: Staining protocol Von Kossa (osteogenic differentiation).

Time / cycles	Work step	Chemical
2x	Washing	DPBS w/o Ca^{2+}/Mg^{2+}
30 min	Fixation	ROTI®Histofix 4 %
2x	Washing	DI water
45 min,<br>under light exposure	Staining of calcium deposits	Silver nitrate (5%)
3x	Washing	DI water
3 min	Reduction by pyrogallol	Pyrogallol (1%)
3x	Washing	DI water
2 min	Fixation	Sodium thiosulfate (5%)
3x	Washing	DI water

Table S3: Staining protocol hematoxylin-eosin (overview staining).

Time / cycles	Work step	Chemical
1x	Washing	Aqua dest.
6 min	Nuclear staining	Hematoxylin solution
15 min	Washing (Bluing)	Running tap water
2 min	Washing	Aqua dest.
3 min	Counterstain	Eosin solution
1x	Washing-up	Tap water
1x	Dehydrating	Increasing alcohol concentration (70%, 96%, 100% ethanol)
1x	Dehydrating	Xylol
1x	Mounting	Coverquick 2000 QPath Mounting Media

Table S4: Staining protocol for alcian blue – nuclear fast red in accordance with "Staining Kit: Alcian Blue - Nuclear Fast Red for Acidic Mucosubstances" (Morphisto GmbH, Offenbach am Main, German, Article no.: 13416) (chondrogenic differentiation).

Time / cycles	Work step	Chemical
3 min	Pretreating	Acetic acid (3%)
30 min	Staining	Alcian blue (1%)
3 min	Differentiation	Acetic acid (3%)
10 min	Washing	Aqua dest.
5 min	Counterstain	Nuclear fast red (0,1%)
1 min	Washing	Aqua dest.
2x 2 min	Dehydrating	96% ethanol, denatured with 1% methyl ethyl ketone (MEK)
2 min	Dehydrating	Isopropanol (2-propanol)
2x 5 min	Dehydrating	Xylol
1x	Mounting	ProTaqs® PARAmount (w/o Xylene, Toluol)
