# Supplementary material for: Multilineage Differentiation Potential of Equine Adipose-Derived Stromal/Stem Cells from Different Sources
Source: Animals (Basel). 2023 Apr 15;13(8):1352. doi: 10.3390/ani13081352 (PMC10135324; doi:10.3390/ani13081352)
Supplement: Supplementary file 1 [file animals-13-01352-s001.zip › Supplementary Table S5_List of primers.pdf]

Supplementary Table S5: List of primers used for SYBR Green RT-qPCR in the cardiomyogenic induction experiments.

Gene	GeneBank/ RefSeq source	Gene ID	Sequence (5'-3')	Exon No	Intron Length	Product Length
ACTB*	NM_001081838.1	100033878	For: GCCAACCGCGAGAAGATGAC	2	448	124
			Rev: AGTCCATCACGATGCCAGTG	3		
GAPDH*	NM_001163856.1	100033897	For: AAGAAGGTGGTGAAGCAGG	9	86	116
			Rev: GCATCGAAGGTGGAAGAGTGGG	10		
RN18s*	NW_019643269.1	100861557	For: ACTCACACGGGAAACCTCAC	1	0	122
			Rev: AACCAGACAAATCGCTCCAC	1		
OCT4/ POU5F1*	XM_001490108	100050785	For: AGCAATTTGCCAAGCTCC	2	633	235
			Rev: GTCTCTGCTTTGCATATCTCC	3-4		
GATA4*	XM_023636259.1	100065126	For: CAGAAAACGGAAGCCAAAGAAC	4	2747	218
			Rev: ACATCGCACTGACCGAGAAC	6		
TNNI3*	NM_001081904.1	100034065	For: TGGATGAGGAGAGATACGATG	6	547	101
			Rev: CTTAAACTTGCCCCGAAGG	7		
MYH6*	XM_023622391.1	111767446	For: GCGCATCGAGTTCAAGAAG	18	1254	188
			Rev: TGATACGCCCAAACTCCTCC	19		
MYH7*	NM_001081758	791234	For: TGAGAAGGGCAAAGGCAAG	15	385	129
			Rev: ATGATGCAACGCACGAAG	16		
DNMT3B	XM_023626323.1	100053900	For: ACTTGGTGATTGGTGGAAGC	18	336	221
			Rev: ACACTCCAGGAACCGAGAGA	19		
MYC*	XM_001497991.1	100068097	For: CAGCGACTCTGAAGAAGAAC	1	1069	241
			Rev: ACTGTCCAACTTAGCCCTC	2		
NKX2-5*	XM_005614765.3	100069632	For: AAGGACCCTCGAGGCGATAA	1	1508	247
			Rev: ACCAGATCTTGACCTGCGTG	2		
MYF6*	NM_001317257.1	100050603	For: CAGCTACAGACCCAAGCAAGA	1	539	202
			Rev: AGGAGAGTTTGCGTTCCTCC	3		

* Already used in: *Trachsel, D. S., H. J. Stage, S. Rausch, S. Trappe, K. Söllig, G. Sponder, R. Merle, J. R. Aschenbach and H. Gehlen (2022): Comparison of Sources and Methods for the Isolation of Equine Adipose Tissue-Derived Stromal/Stem Cells and Preliminary Results on Their Reaction to Incubation with 5- Azacytidine. Animals 12: 2049.*
