# Supplementary material for: Multilineage Differentiation Potential of Equine Adipose-Derived Stromal/Stem Cells from Different Sources
Source: Animals (Basel). 2023 Apr 15;13(8):1352. doi: 10.3390/ani13081352 (PMC10135324; doi:10.3390/ani13081352)
Supplement: Supplementary file 1 [file animals-13-01352-s001.zip › Supplementary Table S6_Cell counts and population doubling time.pdf]

Supplementary Table S6: (A) The cell counts measured by a TC20™ Automated Cell Counter (Bio-Rad Laboratories GmbH, Feldkirchen, Germany) and (B) population doubling times (PDT).

(A)

Cell count [× 10^3; mean ± standard deviation]						
	**abd-ASCs-EXP**	**abd-ASCs-SVF**	**rb-ASCs-EXP**	**rb-ASCs-SVF**	**sc-ASCs-EXP**	**sc-ASCs-SVF**
**day 0**	seeding 30/well					
**day 3**	156 ± 143	98 ± 77	60 ± 33	82 ± 65	124 ± 86	102 ± 83
**day 4**	202 ± 218	174 ± 120	81 ± 20	138 ± 84	125 ± 68	133 ± 121
**day 5**	332 ± 199	326 ± 109	140 ± 31	223 ± 108	360 ± 222	366 ± 202
**day 6**	646 ± 427	417 ± 59	211 ± 94	335 ± 227	453 ± 369	423 ± 330
**day 7**	815 ± 609	443 ± 124	235 ± 177	314 ± 188	456 ± 372	609 ± 460
**day 8**	770 ± 359	451 ± 165	305 ± 163	404 ± 197	533 ± 350	482 ± 156

(B)

Population doubling times (in h) [mean ± standard deviation]						
	**abd-ASCs-EXP**	**abd-ASCs-SVF**	**rb-ASCs-EXP**	**rb-ASCs-SVF**	**sc-ASCs-EXP**	**sc-ASCs-SVF**
**day 3**	25.5 ± 5.5	40.0 ± 17.6	31.7 ± 19.7	41.2 ± 14.0	30.9 ± 6.6	65.0 ± 33.9
**day 4**	33.5 ± 14.3	43.5 ± 13.2	71.3 ± 21.4	35.2 ± 6.2	37.9 ± 0.6	40.2 ± 15.9
**day 5**	41.4 ± 20.7	35.7 ± 5.4	53.6 ± 8.7	46.1 ± 19.0	37.3 ± 12.3	34.3 ± 6.9
**day 6**	37.6 ± 16.0	39.1 ± 2.5	53.9 ± 14.0	52.9 ± 30.4	41.6 ± 11.8	41.7 ± 11.5
**day 7**	44.0 ± 22.4	43.4 ± 4.5	66.4 ± 23.1	55.6 ± 21.0	48.2 ± 13.2	41.9 ± 10.6
**day 8**	42.5 ± 6.5	51.2 ± 7.3	61.4 ± 14.3	56.2 ± 17.9	51.0 ± 12.3	50.3 ± 8.8
**total days 3–8**	40.8 ± 16.6	42.4 ± 5.4	58.4 ± 13.3	52.3 ± 22.4	43.5 ± 12.0	46.2 ± 13.9
